# The Mineral Springs of Michigan

**Published:** 1871-08

**Authors:** 


*The Mineral Springs of Michigan. Report of Committee to the Michigan State Medical Society.*

The committee was composed of Dr. H. O. Hitchcock, of Kalamazoo, Dr. Kedzie, of Lansing, and Dr. Duffield, of Detroit.

The work had been subdivided and assigned in the following manner:

1. The geology and natural history of the mineral wells—Dr. Duffield.

2. The magnetic properties of the waters—Dr. Kedzie.

3. The actual therapeutic value of the springs as shown by the statistics of cases treated by the waters—Dr. Hitchcock.

Dr. Kedzie presented the report for himself and Dr. Hitchcock.

The investigations of Dr. Hitchcock showed that though many hundred patients had visited these springs, the number of cases in which the patients themselves had acknowledged benefit were too few to safely reason from. Dr. Hitchcock had prepared a careful and accurate report of these cases, from which it appeared that while some had been apparently made worse by the drinking of these waters, there was some reason to believe that some cases of neuralgia and paralysis had been benefited, but the query was propounded whether these and other diseases treated at these springs could not be as well treated by a judicious attention to diet and bathing at home.

Dr. Kedzie's report of his investigation as to the magnetic properties of the waters of these wells is an able and conclusive one.

He began by reviewing the history of magnetic wells in the State, and showed that since the first excitement which arose over the supposed discovery of such properties in the water of an artesian well in St. Louis, Gratiot county, these wells had multiplied until there was scarcely a town of any pretension in the State but possessed one or more of these wells. He says: "It becomes therefore a matter of some importance to thoroughly examine this question, for if, contrary to the generally received opinion of scientific men, water is susceptible of magnetic influence, becoming magnetic and imparting magnetic properties to other bodies — and if healing powers reside in the waters of our State arising from this magnetic property, it should be a matter of State pride for our physicians to recognize and develop this new therapeutic agent, and bring it to the notice of the sick and the suffering. But if this magnetic property has no real existence in the water, and is only associated with it by some adventitious circumstances, then the sooner this fact is recognized the more will people be saved from loss and the sick from disappointment cruel as death." The doctor confines himself to the following two questions: "First, Do magnetic properties exist in these waters? Second, Do any healing powers which they may possess arise from this magnetic condition?"

He then reviewed the fanciful notions that had existed in explanation of these supposed properties of water, reviewed the researches of chemists in regard to the magnetic properties of water, among which the most important mentioned are the conclusions of Professors Graham and Faraday.

Since Graham had shown that metallic hydrogen is magnetic, and Faraday had brought to notice the magnetic qualities of oxygen, the question might arise "whether an oxide of hydrogen may not possess magnetic properties." This was rendered the more forcible by the fact that an oxide of iron ($Fe_3 O_4$), called loadstone, possessed this property in such a high degree.

An idea of this kind might have lead some scientific men to favor this notion. A sufficient reply to this is that monoxides do not possess magnetic properties "in any intensity, but only the union of the monoxide and sesquioxide, e. g., $Fe O + Fe_2 O_3 = Fe_3 O_4$, which has received the name of the magnetic oxide. Water is a monoxide, hydrogen forms no sesquioxide, and a mono-sesquioxide is, therefore, impossible."

He then further reviewed the experiments of Becquerel and others, which showed conclusively that water was not magnetic, and says, in comparison with these masterly investigations, "the experiments of individuals made here and there with apparatus obtained from the village blacksmith shop or drug store, made in complete ignorance of many of the laws of magnetic induction, sink into utter insignificance. But this is not the age of respect for authority, and the principles evolved by such masters in science as Michael Faraday 'pale their ineffectual fires' before the confident assertion of some rustic who has tried the experiment, and *knows* the water of his well is magnetic, and magnanimously pities the ignorance and conceit of scientific men who are disposed to accept the authority of Becquerel and Faraday, and doubt the assertion of the rustics."

The doctor then enumerated experiments of his own, showing that the water of these springs possesses no such magnetic properties; that the mistake had arisen from the magnetism possessed by the iron tubing of the well which resulted from its vertical position. He showed that the iron rod that had been supposed to become magnetic by being placed vertically in a vessel of this water, would assume the magnetic property equally as quick if rain water, or *even no water* was used.

So far as he had been able to ascertain, the magnetic power manifested by these tubes in the so-called magnetic wells was proportional to the mass of iron, and varied with the depth and size of the pipe. It is manifested by the attempted artesian well in Charlotte, which has not reached flowing water, but is only a vertical iron tube sunk in the soil, yet it is magnetic just the same as the flowing wells.

The doctor met the assertion that bodies had been magnetized by the water of these wells when removed from these tubes, by experiments made by himself that proved to the contrary.

To illustrate the evidence upon which magnetic properties had been asserted to exist in the waters of these wells, he relates the following amusing incident: "I received, last year, a letter from an intelligent and enterprising editor in this State, saying that I had certainly fallen into error in asserting that the iron tube alone, and not the water, was magnetic. As a triumphant proof of my error, he inclosed a piece of knitting needle, which, before the experiment was made, had been tested and found to be possessed of no magnetic property, yet this needle had been so highly charged by holding it in the water, never coming in contact with the tube at all, that it would readily suspend a shingle nail, and to complete the proof the nail was also inclosed, and the needle certainly did support the nail with ease. As this looked like a pretty strong case, I visited the locality to see the experiment repeated. We armed ourselves with pieces of steel wire reasonably free from magnetism.

"The editor proceeded to repeat his experiment by holding the wire *in the water inside the pipe* from which it was flowing. I objected that this was not a fair test of the magnetic properties of the water, because the tube was itself strongly magnetic, and by placing the needle inside this tube, only an inch in diameter, it was brought within half an inch on every side of a magnetic body, and that magnetic induction will take place through water just as it will through any non-magnetic body. He said this was the way the other needle was charged, but that it made no difference, the water would charge just as well when entirely removed from the tube.

"I suggested placing the water in non-metallic (glass) vessels, and then to try the experiment. He cheerfully accepted the challenge, and he and his friends churned their wires in the water till darkness put an end to the trial without developing a particle of magnetism. He was not convinced, would try the experiment again when he had more time, and let me know the result. Nearly a year has passed, but I have heard nothing further from him on the subject of magnetic water."

The doctor then mentioned that many intelligent persons were deceived by the fact that magnetism may be imparted to pieces of steel, as a knife blade, if it be left in the direction of the magnetic dip or approximating to it, so that at a distance of several feet, or rods even, from the tube, he dips his knife blade in the stream and tests it after it has been thus exposed, he will find it possessed of faint magnetic power. He is told that if he will leave it longer, the water will impart to it its magnetic power more strongly, and it will become more sensibly magnetic. "Is not this *proof?* shall he not believe his own eyes? for he has seen all. Yet not all; for, while he watched, unseen forces have pulled at that bit of steel; gravitation has strained at it with a steady tug, yet all unseen. If you suggest this, the man will grant it at once. But other forces have been at work.

"The magnetic lines of force, like silken cords, have given their invisible and intangible strain upon that bit of steel. Little by little, atom by atom, this eternal force has worked its way through that bit of steel, bringing one molecule after another under its sway; give it time, and it will bring it all under its mysterious control. The force was none the less real because invisible; and he who will reject, even in physical science, the influence of the unseen because it is invisible, will find that the seen and the tangible will only mock and deceive him."

The doctor closed his able report with the conclusion that if the waters possessed therapeutic properties, it was not due to magnetism, for *the water has no magnetism.*

Dr. Duffield read but a small portion of his report.

He stated that we had no mineral springs in this State; that many of the wells termed magnetic have never reached the rock, but that the mineral substances found in the water had been taken up as it filtered through the soil, on its way to some hard and impervious strata upon which it had gathered. Some of these waters contained a large amount of organic matter, which alone should deter the practitioner from prescribing them for his patient. The doctor then gave a grouping of the wells, the principal of which are chalybeate, acidulous or carbonated, sulphurous, and saline. He said, that in prescribing these waters, the physician must remember that his patient must be sent away from the comforts of home, and we cannot be too careful how we send patients to these different springs, lest some time in some graveyard we may meet upon a tombstone the same lament which was found in England:

"Here lies I and my two daughters,
Killed by the drinking of Cheltenham waters;
If we had stuck to Epsom salts,
We would not have been in these here vaults."

